# Correlation between changes in substructure diffusion tensor imaging and neurocognitive outcomes for pediatric brain tumor survivors

**DOI:** 10.1093/noajnl/vdag081

**Published:** 2026-04-03

**Authors:** Ryan T Oglesby, Leslie Chang, Elizabeth Olatunji, Jill S Chotiyanonta, Yuto Uchida, Kengo Onda, Junghoon Lee, Chathurangi H Pathiravasan, Kenichi Oishi, Rachel K Peterson, Sahaja Acharya

**Affiliations:** Department of Radiation Oncology and Molecular Radiation Sciences, Johns Hopkins Medicine, Baltimore, Maryland, USA; Department of Radiation Oncology, University of Minnesota, Minneapolis, Minnesota, USA; Department of Radiation Oncology and Molecular Radiation Sciences, Johns Hopkins Medicine, Baltimore, Maryland, USA; Department of Radiology and Radiological Science, Johns Hopkins Medicine, Baltimore, Maryland, USA; Department of Radiology and Radiological Science, Johns Hopkins Medicine, Baltimore, Maryland, USA; Department of Radiology and Radiological Science, Johns Hopkins Medicine, Baltimore, Maryland, USA; Department of Radiation Oncology and Molecular Radiation Sciences, Johns Hopkins Medicine, Baltimore, Maryland, USA; Department of Biostatistics, Johns Hopkins Bloomberg School of Public Health, Baltimore, Maryland, USA; Department of Radiology and Radiological Science, Johns Hopkins Medicine, Baltimore, Maryland, USA; Department of Neurology, Johns Hopkins Medicine, Baltimore, Maryland, USA; Department of Neurop­sychology, Kennedy Krieger Institute, Baltimore, Maryland, USA; Department of Psychiatry and Behavioral Sciences, Johns Hopkins Medicine, Baltimore, Maryland, USA; Department of Radiation Oncology and Molecular Radiation Sciences, Johns Hopkins Medicine, Baltimore, Maryland, USA

**Keywords:** diffusion tensor imaging, neurocognition, pediatric brain tumor, survivorship, white matter

## Abstract

**Background:**

Central nervous system (CNS) tumors are the second most common pediatric malignancy and the leading cause of cancer-related mortality in children. Although advances in therapy have improved survival, survivors experience neurocognitive decline related to tumor location, surgery, and radiation. Understanding how white matter integrity—assessed by diffusion tensor imaging (DTI)—relates to neurocognitive outcomes is essential for developing treatment-sparing strategies that preserve long-term function.

**Methods:**

This retrospective study included 171 neurocognitive assessments and at least 2 time-matched DTI scans from 68 pediatric brain tumor patients. Healthy controls (*n* = 80) were drawn from the Pediatric Imaging, Neurocognition, and Genetics repository. Axial diffusivity (λ_‖_), radial diffusivity (λ_⊥_), mean diffusivity (MD), and fractional anisotropy (FA) were quantified across 168 neuroanatomical substructures. Temporal changes in DTI were modelled using linear fits, and associations with Wechsler Full Scale Intelligence Quotient (IQ), Working Memory Index (WMI), and Processing Speed Index (PSI) were analyzed using Pearson’s correlation.

**Results:**

Longitudinal assessment revealed median annual declines in IQ and WMI. Changes in white matter diffusivity significantly correlated with neurocognitive outcomes, particularly within the cerebellum, cerebellar and cerebral peduncles, thalamus, and corpus callosum. Increases in λ_‖_ and MD were associated with declines in IQ, WMI, and PSI, while increases in FA correlated with improved WMI.

**Conclusion:**

Loss of white matter integrity was associated with neurocognitive decline in pediatric brain tumor survivors. These findings support the development of interventions aimed at preserving brain function and long-term quality of life by monitoring and mitigating factors associated with neurocognitive change.

Key Points

- Increased white matter diffusivity in the cerebellum, cerebellar and cerebral peduncles, thalamus, and corpus callosum is associated with neurocognitive decline in pediatric brain tumor survivors.

Importance of the Study

This study advances personalized treatment planning aimed at preserving neurocognitive function in pediatric brain tumor survivors. It provides quantitative evidence linking changes in white matter integrity—measured by diffusion tensor imaging (DTI)—to neurocognitive outcomes including intelligence functioning, working memory, and processing speed. The analysis draws from a relatively large cohort, encompassing 171 neurocognitive assessments with time-matched DTI scans from 68 survivors. Findings highlight the vulnerability of critical neuroanatomical structures—cerebellum, cerebellar and cerebral peduncles, thalamus, and corpus callosum—to microstructural changes associated with neurocognitive alterations. These results identify potential neuroanatomical targets for intervention and support integrating DTI-derived biomarkers into individualized treatment planning to optimize neuroprotection and improve long-term quality of life for children with brain tumors.

Second to leukemias, central nervous system (CNS) tumors are the most common pediatric malignancy and the most common solid tumor in children.^1^ The average annual age-adjusted incidence rate of brain and other CNS tumors (both malignant and non-malignant) diagnosed in the United States between 2012 and 2016 for persons aged 0-14 years is 5.74 per 100,000.^2^ The average annual age-adjusted mortality rates (AAMR) of malignant brain and other CNS tumors in the United States between 2012—2016 for persons aged 0—14 years is 0.72 per 100,000.^2^ Treatment has improved with our expanding knowledge of CNS tumors due to advancements in the fields of quantitative imaging, molecular biology, and genetics. Diagnoses are more accurate, surgical intervention is safer, radiation therapy is delivered with greater precision, and chemotherapy is better tolerated.^3^ As a result, the overall 5-year survival of pediatric CNS tumors has increased from an estimated 59% in 1975 to 81% in 2020,^4^ highlighting the need to improve quality of life during the survivorship period.

Survivors of pediatric CNS tumors are at risk of cognitive, educational, social, and physical challenges resulting from the disease and treatment.^5^ Neurocognitive impairment in children with brain tumors—measured by a reduction in intelligence quotient (IQ)—has been correlated with multiple risk factors including tumor location, obstructive hydrocephalus, age at diagnosis, sex, surgical complication, and dose/volume of irradiation.^6^ Cranial radiation, while effective for tumor control, is a major contributor to long-term neurocognitive decline due to its effect on normal brain development, white matter maturation, and neurogenesis.^7^ These effects are most pronounced in younger children and those exposed to higher radiation doses or larger volumes. Currently, our mechanistic understanding of radiation-induced cognitive impairment is incomplete. However, a multifactorial origin is hypothesized in which inflammation contributes to subtle morphological and functional changes by disrupting neurogenesis, reducing white matter integrity (demyelination), and adversely affecting progenitor cells and repair mechanisms.^8^

The pre-frontal cortex, hippocampus, and corpus callosum play a major role in the neurocognitive functions of attention/working memory, learning and memory, and processing speed.^9^ These neuroanatomical structures related to cognitive function are sensitive to the effects of radiation-induced cognitive impairment. As a result, much effort has already been applied to dose sparing of the hippocampus, corpus callosum, and frontal white matter to alleviate the effects of radiation-induced cognitive impairment.^10^ Additionally, damage to the white matter tracts ­connecting the cerebellum to the cortex—specifically the cerebello-thalamo-cortical (CTC) and cortico-ponto-cerebellar (CPC) pathways—have been associated with neurocognitive deficits in pediatric medulloblastoma patients.^11^

To fully understand the mechanisms of neurocognitive impairment in pediatric brain tumor patients, the white matter integrity of each patient should be evaluated over the course of treatment. Arguably, the most effective method of evaluating white matter integrity is with quantitative magnetic resonance imaging. Specifically, diffusion tensor imaging (DTI), derived from diffusion-weighted imaging (DWI), is a specialized modeling technique that provides additional information about how water molecules diffuse differently depending on tissue type, integrity, architecture, and the presence of barriers.^12^ DTI quantifies metrics such as mean diffusivity (MD), axial diffusivity (λ_∥_), radial diffusivity (λ_⊥_), and fractional anisotropy (FA), which together describe white matter organization and integrity.

It has previously been shown that DTI metrics demonstrate a statistically significant correlation with a variety of neurocognitive measures (intelligence, memory, language, executive function, and visual-spatial processing) in healthy older adults,^13^ patients diagnosed with frontotemporal dementia,^14^ Alzheimer’s disease,^15^ traumatic brain injury,^16^ essential tremor,^17^ and cerebral white matter lesions.^18^ Some early work has investigated the relationship between CNS tumors, DTI, and neurocognitive outcomes,^19–21^ however with respect to treatment, these studies have yet to sufficiently advise radiation oncologists who are pursing substructure informed treatment planning in the pediatric population. Data identifying the neuroanatomical substructures most closely associated with neurocognitive changes may inform surgical and radiation planning in future studies. Therefore, the goal of this study was to quantify the correlation between white matter integrity and neurocognition in pediatric brain tumor patients in order to define the neuroanatomical substructures most closely associated with neurocognitive change.

## Methods

### Patient Population

The Institutional Review Board at Johns Hopkins University and Kennedy Krieger Institute approved the retrospective analysis of neuroimaging data and neurocognitive outcomes for this study. Patients (<18 years old) diagnosed with a brain tumor between 2001 and 2022 at our institutions and underwent at least 2 neurocognitive assessments with 2 time-matched DTI sessions were eligible for inclusion in this study. Patient image sets were excluded from this study if the time between image acquisition and neurocognitive assessment was greater than 6 months, or if significant motion or inhomogeneity artifacts were observed on the DTI, such as those caused by a surgical resection cavity or drainage device.

### Healthy Participants

Healthy participants were selected from the Pediatric Imaging, Neurocognition, and Genetics (PING) data repository.^22^ The PING data repository contains multimodal neuroimaging, developmental histories, behavioral and cognitive assessments, and whole genome genotyping for 1493 healthy children and adolescents ranging from 3 to 21 years of age. Exclusion criteria included: neurological disorders; history of head trauma; preterm birth (<36 weeks); diagnosis of an autism spectrum disorder, bipolar disorder, schizophrenia, or intellectual disability; pregnancy; and daily illicit drug use by the mother for more than one trimester. DTI data from 80 participants (40 female; 40 male) were selected from the PING data repository with age linearly spaced between 2 and 20 years old. These data were used to characterize the anticipated change in substructure diffusivity in a healthy developing brain. Imaging in the PING dataset was acquired at multiple sites using 3T GE, Siemens, and Philips scanners with harmonized acquisition protocols to ensure comparable quantitative diffusion measures.^22^

### MRI Acquisition

Given that the retrospective data analyzed for this study was acquired over the duration of 20+ years, MRI scanners and sequences used to collect images differed marginally in their hardware and protocol. Efforts were made to homogenize the image datasets according to the following inclusion criteria: vendor = Siemens (Siemens Healthineers), B_0_ = 1.5 or 3T, b-value  =  800 or 1000 s/mm^2^, and voxel size  =  1.25 × 1.25 × 2.5 cm^3^. For each patient, the same field strength and b-value were used across all longitudinal evaluations. The Siemens MRI scanners used in this study for image acquisition included: Skyra (3T), Prisma (3T), Verio (3T), Trio (3T), Sola (1.5T), Aera (1.5T), Avanto (1.5T), and Espree (1.5T).

### MRI Analysis

The DWIs were processed using MRICloud software.^23^ Image pre-processing steps included de-identification, linear eddy current, and motion correction. A least squares-based tensor fitting was applied, incorporating the Geman-McClure M-estimator^24^ and corrected inter-slice intensity discontinuity weighting terms for pixel-by-pixel outlier rejection.^25^ DTI derived scalar measures (λ_‖_, λ_⊥_, MD, and FA) were calculated from the tensor fitting. A multi-contrast multi-atlas likelihood-fusion algorithm was used to automatically parcellate the brain into 168 neuroanatomical substructures based on the DTI data.^26^ Axially symmetric structures were averaged (left and right) to simplify analysis. The auto-parcellation algorithm implements a MD threshold of 0.0065 mm^2^/s above which a voxel may be ignored and classified as cerebrospinal fluid. Tumors were manually contoured in 3D Slicer (v5.2.2; slicer.org)^27^ by a radiation oncologist with more than 8 years of clinical experience, and all parcellated neuroanatomical substructures were reviewed under the supervision of a neuroradiologist with more than 25 years of experience in brain MRI research. Tumor volumes were subtracted from overlapping automatically parcellated substructures. The volumes and mean DTI derived scalar measures for each neuroanatomical substructure were then exported from 3D Slicer for statistical analysis.

### Neurocognitive Assessment

Neurocognitive assessment of each patient included Full Scale Intelligence Quotient (IQ), Working Memory Index (WMI), and Processing Speed Index (PSI). Full scale IQ, representing the overall intellectual functioning, was evaluated using the Wechsler Intelligence Scale for Children, Fourth (WISC-IV) or Fifth (WISC-V) Edition or the Wechsler Adult Intelligence Scale, Fourth Edition (WAIS-IV) depending on the patient’s age at assessment. The WMI consisted of the Digit Span, Picture Span, Arithmetic, and/or Letter-Number Sequencing subtests depending on which Wechsler version was administered. The Coding and Symbol Search subtests comprised the PSI. Neurocognitive scores for IQ, WMI, and PSI are age-corrected, norm referenced with a mean of 100 and standard deviation of 15. The proportion of patients with complete neurocognitive datasets (at least 2 timepoints for IQ, WMI, and PSI) was 75%. Partially complete neurocognitive evaluation was typically due to clinical necessity or inability to complete specific tasks (eg, impaired motor function, remote test administration during the COVID-19 pandemic).

### Statistical Analysis

Longitudinal change in substructure DTI and neurocognitive measures were quantified by the slope of a least squares linear fit for each patient. The correlation between change in substructure DTI and neurocognitive measures were quantified by a Pearson correlation coefficient. Outliers greater than 1.7 times the interquartile range (IQR) were excluded from correlation analysis. A conservative Bonferroni correction was applied by dividing the *P* value by a factor of 5 to reduce the likelihood of false positives when evaluating multiple comparisons on the same dataset.^28^ Figures were generated and statistics computed using MATLAB (R2022b, The MathWorks).

## Results

### Patient Population

A total of 68 patients (47.1% female) with a median age at diagnosis of 7.4 (IQR = 5.9; range = 0.3-17.2) years old met eligibility criteria. Brain tumor types included low-grade glioma (36.8%), high-grade glioma (4.4%), medulloblastoma (23.5%), craniopharyngioma (7.4%), ependymoma (11.8%), germ cell tumor (7.3%), and other tumor types (8.8%). Treatments included surgery, radiation therapy, and/or chemotherapy. All but 4 patients underwent some form of surgery: gross total resection (55.9%), subtotal resection (19.1%), or biopsy (19.1%). The median time interval between surgery and the first follow-up DTI used for analysis was 398 days (IQR = 554 days). Radiation therapy was administered in 57.4% of patients with a median dose of 54 Gy; 32.4% received craniospinal irradiation and 25% received focal irradiation. The median time interval between completion of radiation therapy and the first follow-up DTI used for analysis was 233 days (IQR = 372 days). Chemotherapy was utilized in 64.7% of patients. Patient demographics and clinical characteristics are summarized in Table 1. Overall, the cohort represented a heterogeneous but typical pediatric brain tumor population, with diverse diagnoses and treatment exposures reflecting varying risks for neurocognitive and white matter change.

**Table 1.** Pediatric brain tumor patient demographics and clinical characteristics

Characteristic	Category	Patient cohort	(*n* = 68)
Sex	Female	32	(47.1%)
	Male	36	(52.9%)
Age at diagnosis	0-4	20	(29.4%)
	5-9	28	(41.2%)
	10-14	14	(20.6%)
	15-19	6	(8.8%)
Histology	Low-grade glioma	25	(36.8%)
	High-grade glioma	3	(4.4%)
	Medulloblastoma	16	(23.5%)
	Craniopharyngioma	5	(7.4%)
	Ependymoma	8	(11.8%)
	Germ cell tumor	5	(7.3%)
	Other	6	(8.8%)
Tumor location*	Lobes of the brain	14	(20.6%)
	Suprasellar	16	(23.5%)
	Lateral ventricle	3	(4.4%)
	Pineal gland	5	(7.4%)
	Thalamus	4	(5.9%)
	Cerebellum/fourth ventricle	26	(38.2%)
	Brainstem	6	(8.8%)
Surgery	None	4	(5.9%)
	Biopsy	13	(19.1%)
	Subtotal resection	13	(19.1%)
	Gross total resection	38	(55.9%)
Radiation therapy	None	29	(42.6%)
	Focal irradiation	17	(25.0%)
	Craniospinal irradiation	22	(32.4%)
Chemotherapy	None	24	(35.3%)
	Yes	44	(64.7%)

* Patients with diffuse metastatic disease may have been categorized into multiple tumor location categories.

### Neurocognitive Assessment

**Figure 1.**
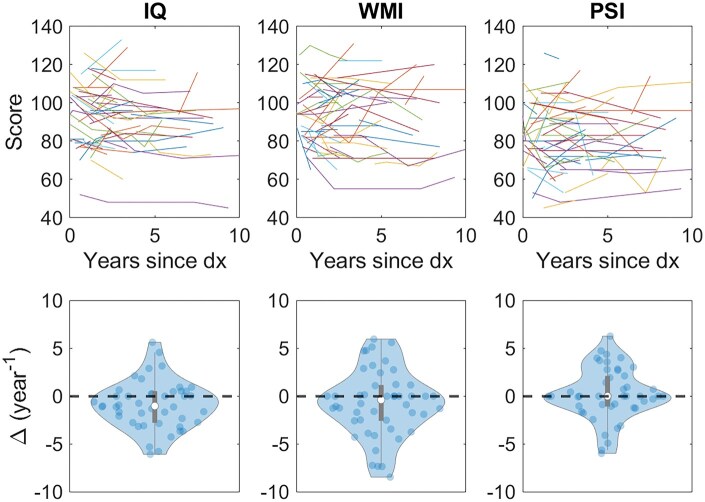
Change in IQ, WMI, and PSI. Raw scores as a function of time (top row) and slope of least squares linear fit representing the change per year since diagnosis (dx) for each patient (bottom row). Intelligence Quotient (IQ), Working Memory Index (WMI), and Processing Speed Index (PSI).

Changes in neurocognitive performance for each patient are illustrated in Figure 1. The standard scores for IQ, WMI, and PSI as a function of time are shown in the top row, and the slopes of the least squares linear fit for each patient are distributed as violin plots in the bottom row. The expected median change in each neurocognitive parameter for a healthy population is zero (dotted line). The median annual change for the pediatric brain tumor patient population was −1.0, −0.4, and 0 per year for IQ, WMI, and PSI, respectively. These results indicate a modest overall decline in IQ and WMI over time, while PSI remained stable across the study cohort.

### Diffusion Tensor Imaging

**Figure 2.**
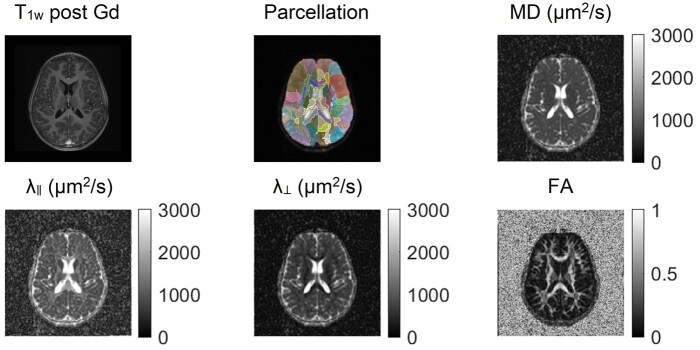
MRI sequences representative of a single patient timepoint. T_1_-weighted post gadolinium (T_1w_ post Gd); parcellation map; mean diffusivity (MD); axial diffusivity (_**λ**__∥_); radial diffusivity (_**λ**__⊥_); and fractional anisotropy (FA). The brain was parcellated into 168 neuroanatomical substructures using the multi-contrast multi-atlas framework described by Tang et al (2014), which defines the full structure set.^26^

**Figure 3.**
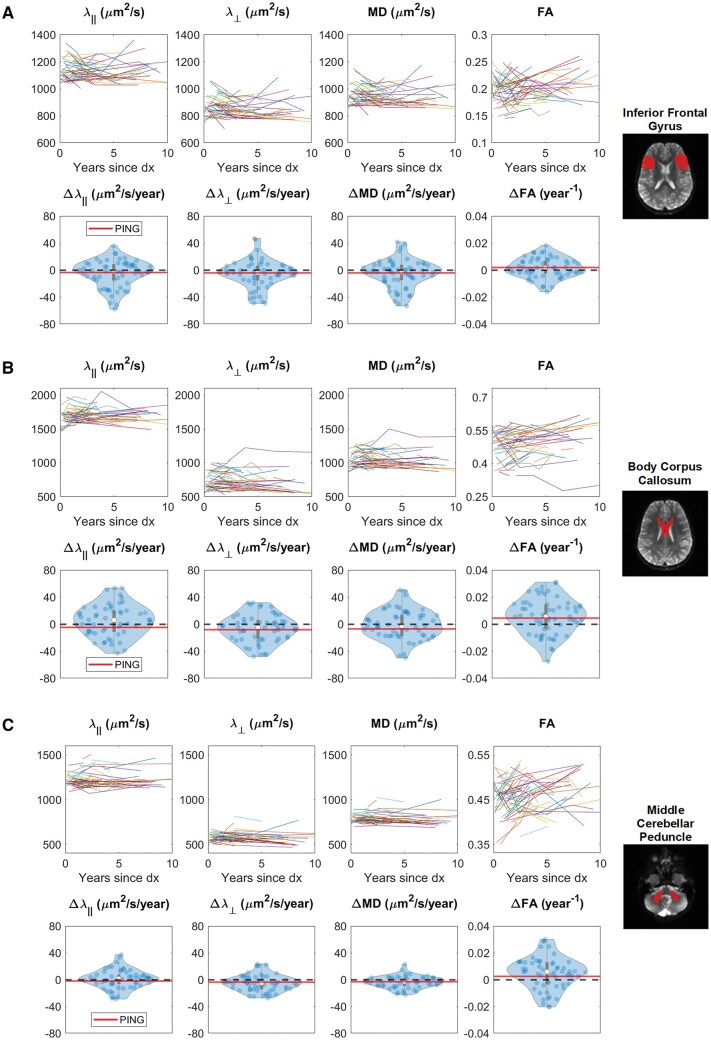
Change in substructure DTI for the (A) inferior frontal gyrus, (B) body of the corpus callosum, and (C) middle cerebellar peduncle. Raw image data as a function of time (top row) and slope of least squares linear fit representing the change per year for each patient (bottom row). Axial diffusivity (_**λ**__∥_); radial diffusivity (λ_⊥_); mean diffusivity (MD); fractional anisotropy (FA); and pediatric imaging, neurocognition and genetics (PING) data repository. For reference, the expected mean change for a healthy developing brain derived from the PING data repository is shown as a solid red line, while a zero-change slope is indicated by a dotted line.

Figure 2 illustrates the MRI sequences evaluated for a single patient timepoint, including a T_1_-weighted post gadolinium structural scan, a parcellation map generated from a pediatric brain atlas, and quantitative DTI maps, including MD, λ_∥_, λ_⊥_, and FA. Figure 3 illustrates the change in each DTI parameter for 3 representative substructures: inferior frontal gyrus (Figure 3A), body of the corpus callosum (Figure 3B), and middle cerebellar peduncle (Figure 3C). The raw change in each DTI parameter as a function of time is shown in the top rows, and the slopes of the least squares linear fit for each patient are distributed as violin plots in the bottom rows.

For reference, the expected mean change in DTI parameter for a healthy developing brain—quantified from the PING data repository—is plotted as a solid red line, with a zero slope shown as a dotted line. Two notable deviations between the healthy developing brain and the pediatric patient population were observed in λ_∥_ in the body of the corpus callosum and FA in the middle cerebellar peduncle. The expected change in λ_∥_ in the body of the corpus callosum from the PING data repository was −5, while the median change in λ_∥_ measured in the pediatric patient population was 6. Similarly, the expected change in FA in the middle cerebellar peduncle from the PING dataset was 0.006, whereas the median change in FA in the pediatric population was 0.002. Collectively, these findings suggest that patients exhibited altered microstructural trajectories compared to healthy developmental trends, particularly in major white matter tracts.

### Correlation Between Neurocognitive Assessment and DTI

**Figure 4.**
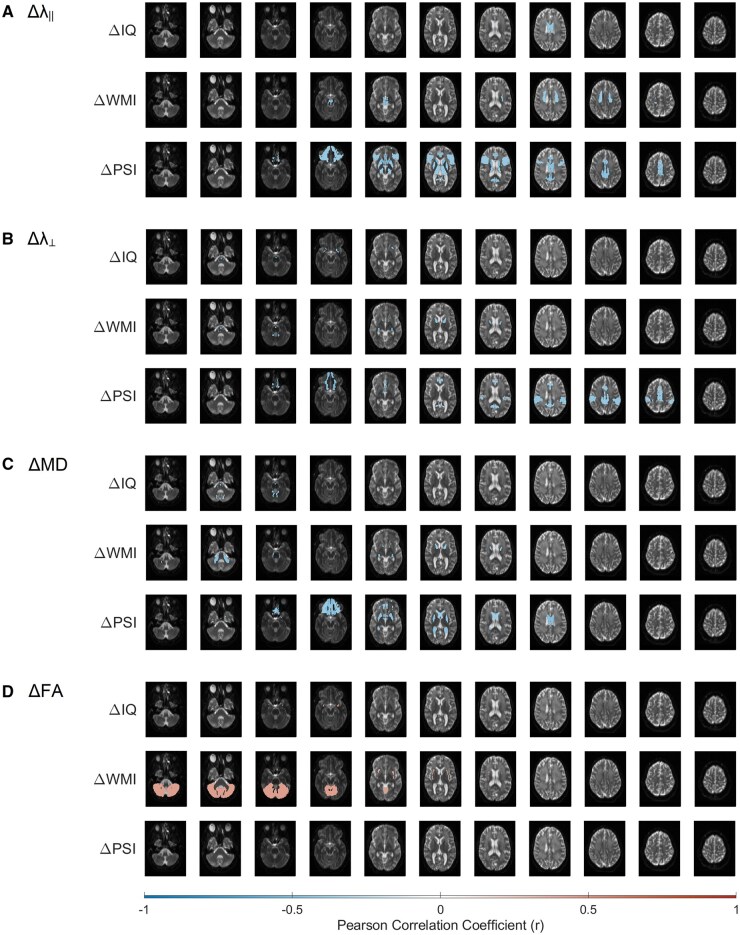
Person’s correlation coefficient (*r*) maps overlaid on the pediatric brain atlas comparing change in neurocognitive assessment and substructure DTI. Significant correlations were plotted if *n* ≥ 3, *r* ≥ 0.3, and *P* ≤ .01. Negative correlations are illustrated in blue and positive correlations in red. (A) Change in axial diffusivity (Δλ_∥_); (B) change in radial diffusivity (Δλ_⊥_); (C) change in mean diffusivity (ΔMD); and (D) change in fractional anisotropy (ΔFA). Neurocognitive measures include change in intelligence quotient (ΔIQ); change in working memory index (ΔWMI); and change in processing speed index (ΔPSI).

Of the 68 pediatric brain tumor patients included in this study, 171 DTI and neurocognitive assessments were examined, with some patients having up to five timepoints. The median time interval between neurocognitive assessment and the corresponding DTI was 42 days (IQR = 64.5 days). Pearson’s correlation coefficients (*r*) were calculated between the change in neurocognitive performance (IQ, WMI, and PSI) shown in Figure 1 and the change in DTI parameters (λ_∥_, λ_⊥_, MD, and FA) shown in Figure 3 for 168 neuroanatomical substructures. Correlation with r > 0.3 and *P* < .01 were overlaid on the pediatric brain atlas in Figure 4. Correlations between neurocognitive outcomes and λ_∥_ are illustrated in Figure 4A, λ_⊥_ in Figure 4B, MD in Figure 4C, and FA in Figure 4D. Negative correlations are illustrated in blue and positive correlations in red.

**Figure 5.**
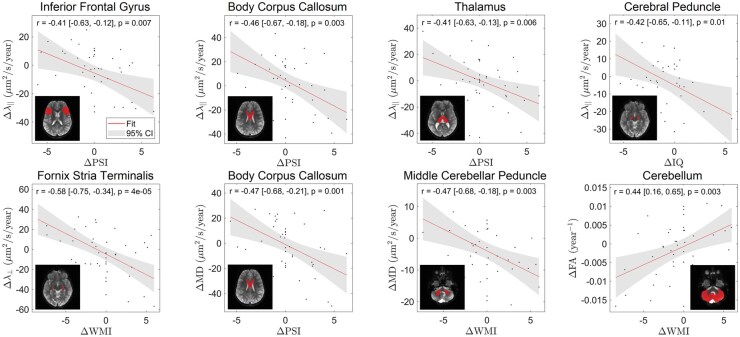
Scatter plots illustrating statistically significant correlations between change in substructure DTI and neurocognitive outcomes per year. Each data point represents an individual patient. The solid line indicates the line of best fit, and the shaded region denotes the 95% CI. Axial diffusivity (λ_∥_); radial diffusivity (λ_⊥_); mean diffusivity (MD); fractional anisotropy (FA); Intelligence Quotient (IQ), Working Memory Index (WMI), and Processing Speed Index (PSI).

Given the large number of substructures, DTI parameters, and neurocognitive outcomes, 8 significant substructure correlations were selected for emphasis in Figure 5. Each data point in Figure 5 represents an individual patient, with the red line indicating the line of best fit and the shaded region denoting the 95% confidence interval (CI). The change in λ_∥_ in the inferior frontal gyrus was negatively correlated with a change in PSI (*r* = −0.41; *P* = .007). The change in λ_∥_ in the body of the corpus callosum was negatively correlated with PSI (*r* = −0.46; *P* = .003). The change in λ_∥_ in the thalamus was negatively correlated with PSI (*r* = −0.41; *P* = .006). The change in λ_∥_ in the cerebral peduncle was negatively correlated with IQ (*r* = −0.42; *P* = .01). The change in λ_⊥_ in the fornix stria terminalis was negatively correlated with WMI (*r* = −0.58; *P* = 4 × 10^−5^). The change in MD in the body of the corpus callosum was negatively correlated with PSI (*r* = −0.47; *P* = .001). The change in MD in the middle cerebellar peduncle was negatively correlated with WMI (*r* = −0.47; *P* = .003). The change in FA in the cerebellum was positively correlated with WMI (*r* = −0.44; *P* = .003). Figures 4 and 5 illustrate the longitudinal change in DTI metrics relative to change in neurocognitive performance across time, rather than cross-sectional associations at a single time point. Including multiple diffusivity measures (λ_∥_, λ_⊥_, and MD), in addition to fractional anisotropy (FA), allows for more detailed interpretation of the underlying microstructural changes, as these measures capture complementary aspects of axonal and myelin integrity.

In addition to those substructures shown in Figure 5, significant correlations were observed in the anterior limb of the internal capsule (Δλ_⊥_ − ΔWMI; *r* = −0.45 [−0.66, −0.17]; *P* = .002), superior corona radiata (Δλ_∥_—ΔWSI; *r* = −0.41 [−0.64, −0.11]; *P* = .009), and inferior fronto-occipital fasciculus (Δλ_⊥_—ΔIQ; *r* = −0.47 [−0.69, −0.18]; *P* = .003). Together, these findings demonstrate that declines in white matter integrity were significantly associated with worsening neurocognitive performance across multiple brain regions.

## Discussion

In this retrospective analysis, we quantified the correlation between change in substructure DTI (λ_∥_, λ_⊥_, MD, and FA) and neurocognitive outcomes (IQ, WMI, and PSI) in 68 pediatric brain tumor patients. The results of this work support the hypothesis that declines in white matter integrity are correlated with worsening neurocognitive performance following brain tumor treatment. Increased white matter diffusivity—indicating greater demyelination—was negatively correlated with decreased IQ, WMI, and PSI. Similarly, increased FA—suggesting greater molecular restriction and healthier tissue—was positively correlated with increased WMI. Importantly, these findings represent global longitudinal trends across a heterogeneous cohort and do not isolate the effects of specific treatment modalities.

Some of the neuroanatomical substructures parcellated in this study, which are part of the CTC and CPC pathways, demonstrate significant correlations between DTI and neurocognitive outcomes. These substructures—shown in Figure 5—include the cerebellum (ΔFA − ΔWMI; *r* = 0.44 [0.16, 0.65]; *P* = .003), middle cerebellar peduncle (ΔMD − ΔWMI; *r* = −0.47 [−0.68, −0.18]; *P* = .003), thalamus (Δλ_∥_ − ΔPSI; *r* = −0.41 [−0.63, −0.13]; *P* = .006), cerebral peduncle (Δλ_∥_ − ΔIQ; *r* = −0.42 [−0.65, −0.11]; *P* = .01), and inferior frontal gyrus (Δλ_∥_ − ΔPSI; *r* = −0.41 [−0.63, −0.12]; *P* = .007). These results are consistent with the literature which describes an association between white matter damage quantified by DTI and neurocognitive impairments in pediatric medulloblastoma patients.^11^ Specifically, with respect to the CTC and CPC pathways, Law et al found that reduced FA and increased λ_⊥_ within the entire CTC pathway predicted lower working memory in pediatric patients with posterior fossa tumors^29^ and compromised cerebrocerebellar circuitry had a mediating effect on working memory following treatment of medulloblastoma.^30^

Another neuroanatomical substructure previously observed to demonstrate correlations between white matter integrity and neurocognitive performance is the corpus callosum. The body of the corpus callosum—illustrated twice in Figure 5—demonstrated significant correlations between Δλ_∥_ − ΔIQ (*r* = −0.42 [−0.65, −0.12]; *P* = .009), Δλ_∥_ − ΔPSI (*r* = −0.46 [−0.67, −0.18]; *P* = .003), and ΔMD − ΔPSI (*r* = −0.47 [−0.68, −0.21]; *P* = .001). These results align with prior literature which described positive correlations between corpus callosum FA and processing speed,^31^^,^^32^ and between corpus callosum FA and motor speed.^33^ Prior studies have also shown that radiation dose is associated with altered FA and MD in the corpus callosum^32^^,^^33^; however, the present study was not designed to isolate treatment-specific effects.

In addition to those neuroanatomical substructures previously reported on in literature, we found significant correlations between DTI and neurocognitive outcomes in the anterior limb of the internal capsule (Δλ_⊥_ − ΔWMI; *r* = −0.45 [−0.66, −0.17]; *P* = .002), superior corona radiata (Δλ_∥_ − ΔWSI; *R* = −0.41 [−0.64, −0.11]; *P* = .009), fornix stria terminalis (Δλ_⊥_ − ΔWMI; *r* = −0.58 [−0.75, −0.43]; *P* = .00004), and inferior fronto-occipital fasciculus (Δλ_⊥_ − ΔIQ; *r* = −0.47 [−0.69, −0.18]; *P* = .003).

The majority of Pearson’s correlation coefficients reported in this work were within the magnitude range of 0.4-0.5 with *P* value ≤ .01. According to Cohen, these correlations represent a moderate effect size,^34^ providing compelling evidence that longitudinal changes in DTI measures reflect measurable neurocognitive alterations over time. While radiation therapy is a known contributor to white matter injury and cognitive decline, this analysis was not limited to irradiated patients and therefore captures aggregate effects of multiple treatment and disease-related factors. Consequently, all conclusions are restricted to observed population-level associations rather than direct treatment causation. A planned subanalysis will examine how radiation exposure, tumor location, and treatment modality influence these relationships, enabling stronger causal inferences in future work.

The retrospective nature of this investigation presents several inherent limitations: (1) inhomogeneous image acquisition parameters due to the rarity of pediatric brain tumors and the evolving MRI technology over more than 20 years of data collection; (2) MR field inhomogeneities causing artifacts near patient resection cavities, with affected structures manually excluded from analysis; (3) expected DTI changes in the developing brain, with longitudinal changes qualitatively compared to the healthy PING dataset; (4) heterogeneous tumor location and histology, which may introduce potential correlation bias (eg, 38% of the cohort had tumors in the cerebellum or fourth ventricle regions, potentially skewing neurocognitive correlations toward the cerebellum and cerebellar peduncles); (5) imperfect alignment of image acquisition and neurocognitive assessment timepoints, with a median gap of 42 days and a maximum of 6 months; and (6) follow-up timepoints that may be insufficient to fully capture long-term neurocognitive impairments. Additionally, given the large number of comparisons, the possibility of false-positive correlations cannot be entirely excluded despite the application of Bonferroni correction. Future studies with larger, stratified cohorts will be needed to validate these findings.

Future work will focus on subdividing our correlation analysis by clinical characteristics such as sex, age, histology, tumor location, and treatment paradigm to identify whether certain groups exhibit stronger correlations, potentially highlighting subgroups more susceptible to cognitive decline. These planned analyses will be conducted within the ongoing prospective study Cognitive Outcomes After Brain Substructure-informed Radiation Planning in Pediatric Patients (CogRT; ClinicalTrials.gov NCT05658731), which is designed to evaluate longitudinal neurocognitive and DTI changes following radiation therapy in pediatric brain tumor survivors. This work has several important implications for clinical practice, including the potential to inform treatment-sparing protocols, guide serial monitoring of DTI and neurocognition during treatment and follow-up, and support rehabilitation strategies that promote resilience and recovery in pediatric brain tumor survivors.

## Conclusion

This study demonstrates significant correlations between white matter integrity, characterized by DTI, and neurocognitive performance in pediatric brain tumor survivors. Critical neuroanatomical structures, including the cerebellum, cerebellar peduncles, thalamus, cerebral peduncles, and corpus callosum, were identified as particularly associated with longitudinal changes in neurocognitive outcomes. These findings indicate that longitudinal changes in specific substructures of the brain are closely linked to measurable alterations in neurocognitive function. While the present analysis does not isolate the effects of specific treatments, it provides a foundation for future studies to investigate how clinical factors, tumor characteristics, and radiation exposure may influence these relationships. Understanding these correlations can inform the design of prospective studies, such as the ongoing CogRT trial (NCT05658731), and contribute to strategies for monitoring and supporting cognitive function in pediatric brain tumor survivors.

## Data Availability

The datasets generated during and/or analyzed during the current study are available from the corresponding author upon reasonable request.
